# SPARC: a new approach to quantifying gait smoothness in patients with Parkinson’s disease

**DOI:** 10.1186/s12984-018-0398-3

**Published:** 2018-06-18

**Authors:** Yoav Beck, Talia Herman, Marina Brozgol, Nir Giladi, Anat Mirelman, Jeffrey M. Hausdorff

**Affiliations:** 1 Center for the study of Movement, Cognition, and Mobility, Neurological Institute, Department of Neurology, Tel Aviv Sourasky Medical Center, 6 Weizmann street, 64239 Tel Aviv, Israel; 2 Graduate Training Centre of Neuroscience/ IMPRS for Cognitive and Systems Neuroscience, Tübingen, Germany; 3 Neurological Institute, Tel Aviv Sourasky Medical Center, Tel Aviv, Israel; 4 Department of Neurology, Sackler Faculty of Medicine, Tel Aviv University, Tel Aviv, Israel; 5 Sagol School of Neuroscience, Tel Aviv University, Tel Aviv, Israel; 6 Department of Physical Therapy, Sackler Faculty of Medicine, Tel Aviv University, Tel Aviv, Israel; 7 Rush Alzheimer’s Disease Center and Department of Orthopaedic Surgery, Rush University Medical Center, Chicago, USA

**Keywords:** Accelerometers, Parkinson’s disease, Smoothness, Motor control

## Abstract

**Background:**

Impairments in biomechanics and neural control can disrupt the timing and muscle pattern activation necessary for smooth gait. Gait is one of the most affected motor characteristics in Parkinson’s disease (PD), but its smoothness has not been well-studied. This work applies the recently proposed spectral arc length measure (SPARC) to study, for the first time, gait in patients with PD. We hypothesized that the gait of patients with PD would be less smooth than that of healthy controls, as reflected in the SPARC measures.

**Methods:**

The gait of 101 PD patients and 39 healthy controls was assessed using an inertial sensor. Smoothness of gait was estimated with SPARC (respectively from acceleration and angular velocity signals, SPARC-Acc and SPARC-Gyro) and harmonic ratios. Correlations between SPARC, traditional gait measures and the motor part of the Unified Parkinson’s Disease Rating Scale (UPDRS) were evaluated. Measurements and analysis were conducted with and without anti-PD medication.

**Results:**

SPARC measures were lower (less smooth) in PD than in controls (SPARC-Acc: PD: − 6.11 ± 0.74; CO: -5.17 ± 0.79; *p* <  0.001). When comparing PD to controls, SPARC-Acc differed more than other measures of gait (i.e., largest effect size, which was > 1). SPARC measures were correlated with UPDRS motor score (*r* = − 0.65), while they were independent of other measures of gait smoothness. PD gait in the on state was smoother than in the off state (*p* <  0.001).

**Conclusions:**

SPARC calculated from trunk acceleration and angular velocity signals provide valid measures of walking smoothness in PD. SPARC is sensitive to Parkinson’s disease and PD medications and can be used of as another, complementary measure of the motor control of walking in PD.

## Background

Gait is a major component of daily physical activities and a key to functional independence [1]. Gait disturbances are one of the clinical hallmarks of Parkinson’s disease (PD). Among patients with PD, the gait pattern is characterized by reduced speed, short stride length, shuffling steps, and, occasionally, typically in more advanced patients, freezing of gait episodes [2]. These changes in gait lead to disability, restricted function and reduced health-related quality of life.

Although faster gait speeds have been associated with better function [3–5], some aspects of physical function and mobility are not represented by speed but by the quality of movement control [6, 7]; i.e. how hesitant, stable, abrupt or smooth the gait is. The present work addresses estimation of gait smoothness from inertial sensors. Smoothness of gait is a quality that reflects the continuousness or non-intermittency of walking. Measuring walking smoothness aims to quantify intermittency during gait; intermittent movements tend to have many accelerating and decelerating phases.

Several measures have been used to estimate gait smoothness [8–10]. Video motion analysis permit calculation of the smoothness of the limbs movements from the estimated jerk measured at the joints during gait [11, 12]. Accelerometers need a different approach. In [8, 10], gait smoothness was estimated from the mean and maximum anteroposterior acceleration of the lower back. In [9, 10], gait variability (i.e., stride-to-stride fluctuations) is considered a descriptor of smoothness of gait. Indeed, patients with PD have increased stride-to-stride variability [13, 14], compared to healthy controls. Previous studies have also shown that reduced gait stability may predispose individuals with PD to falls [15–20]. However, while gait variability measures stepping arrhythmicity, it is not necessarily a direct measure of the smoothness of gait. Moreover it is of importance to note that jerk [21], mean acceleration or gait variability computation are not totally independent of the movement amplitude or duration, thus compounding interpretation of comparisons between tasks and subjects. In contrast, smoothness can be independent of those factors.

Harmonic ratios are commonly used (HR, the ratios between the sum of the magnitudes of the even to the odd harmonics over a single stride) to measure smoothness of gait [22–24]. They have been shown to differ in PD and controls [25]. However, rather than smoothness, the harmonic ratios may be viewed more as a measure of the symmetry of the movement of the two legs during walking (i.e., gait can be symmetric and not smooth).

Recently, Balasubramanian et al. introduced a novel measure to characterize movement smoothness, based on the spectral arc length [26]. The spectral arc length (SPARC) measures the arc length of the Fourier magnitude spectrum within an adaptive frequency range. This approach addresses limitations of previously proposed measures of smoothness [26, 27]. Indeed, SPARC quantifies movement intermittencies but is independent of its amplitude or duration. The goal of the present work was to study, for the first time, SPARC in the context of gait in PD in order to assess whether it might provide useful information for objective characterization of the gait of patients with PD. Furthermore, the PD cohort was split into fallers and non-fallers to begin to investigate the association of this measure with fall risk. We hypothesized that gait would be less smooth in patients with PD, compared to healthy controls, and, further, that the gait of PD fallers would be less smooth than the PD non-fallers. To better understand this new measure of smoothness in PD, we also examined the effects of anti-parkinsonian medications and explored the correlation and differences between SPARC, harmonic ratios, gait speed, stride time variability, and clinical measures.

## Methods

### Participants and procedures

The present analyses were applied to a dataset previously collected to study white matter changes in PD [28]. Briefly, 101 patients with PD (mean age: 64.5 ± 9.3 yrs., 24 women) and 39 elderly control subjects (mean age: 77.8 ± 3.9 yrs., 25 women) participated in this study. Patients with PD were included if they were diagnosed with PD by a movement disorders specialist, if they were between 40 and 85 years of age, and if they were not demented based on the Mini Mental State Examination (MMSE) [29]. PD patients with more than 1 fall in the previous year were considered fallers, while non-fallers reported no falls in that period. Patients were excluded if they underwent brain surgery in the past, including implanted deep brain stimulation, or had significant co-morbidities likely to affect gait, e.g., acute illness, orthopedic disease, other neurologic disease, or history of stroke. All subjects provided informed written consent as approved by the local human studies committee. The gait task consisted of a 50 m walk with a turn in the middle. Subjects were fitted with a small, lightweight inertial measurement unit (Hybrid, McRoberts, The Hague, Netherlands) placed on the lower back (at the level of lumbar vertebrae 4–5) using an elastic belt. The device includes a triaxial accelerometer (sensor range and resolution: ±6 g and ± mg, respectively) and a triaxial gyroscope (sensor range and resolution: ±100°/second and ± 0.0069°/second, respectively). The signals acquired were 3 acceleration axes, vertical acceleration (V), mediolateral acceleration (ML), anterior posterior acceleration (AP), and 3 angular velocity axes, yaw, pitch, and roll. The signals were recorded on a Secure Digital (SD) card at a sample frequency of 100 Hz, and later transferred to a personal computer for further analysis. The PD subjects were first tested in the OFF medication state, at least 12 h after they took their anti-parkinsonian medications. The examination was repeated in the ON state, about 1 h after taking their first morning dose of their anti-parkinsonian drugs.

### Data processing and spectral arc length

Steps were located from the vertical field of the signal (V) as peaks above an adaptive threshold. The threshold was based on the median of the signal. The algorithm was developed in the lab and tested against an instrumented mat (GAITRite®) and prove to be more accurate than [30]. Turns were identified from the yaw axis as described in [30] and removed from further analysis. We chose to compare SPARC with several of the most common metrics characterizing gait and smoothness of gait in Parkinson’s disease. A summary of the measures calculated can be seen in Table 1. In the original paper [26], SPARC was used to measure smoothness from the velocity profile of a movement. However, as the authors suggested, to measure gait smoothness, SPARC could also be applied to any semi-periodic signal, e.g., acceleration or gyroscope signals during walking. To determine the spectral arc length, the algorithm requires two threshold values. In order to avoid overfitting, the thresholds values were decided from investigation on different cohorts [31]. A grid search, using a logistic regression was performed to decide on the best couple of parameters for the SPARC estimation. A frequency upper threshold that should bound the “normal and abnormal” movement range; i.e., normal and disease altered gait needs to be inside the threshold boundaries. Normal gait in healthy older adults is typically less than 2 steps per second [32]. Therefore, the higher bound threshold selected was ${\omega}_c^{max}=10\pi$ (i.e., 5 Hz). The second value is a lower bound threshold; it controls the trade-off between SPARC sensitivity and noise contamination. Here $\overline{A}=0.01$ was chosen.

**Table 1:** Summary of the gait measures

Gait measures	Description
SPARC(-Acc, -V, -AP, −ML & -Gyro, -Yaw, −Roll, -Pitch)	Spectral arc length (unitless)
Harmonic Ratios (V, AP, ML)	Measure of step to step symmetry (unitless)
Mean Gait Speed	Mean gait speed (m/s)
Stride time mean	Mean stride time (s)
Stride time variability	Coefficient of variation of the stride time (%)

Note: *Acc* acceleration, *V* vertical, *AP* anteroposterior, *ML* medio-lateral, *Gyro* gyroscope

In short, the calculation of SPARC is performed from the following steps (more details in [27]):

- Step 1: Select the walking boots (i.e., remove turns).
- Step 2: Compute the spectrum for each acceleration bout.
- Step 3: Normalize each spectrum.
- Step 4: Calculate SPARC for each walking bout.
- Step 5: Average SPARC over all the walking bouts.

$$ SPARC=-{\int}_0^{\omega_c}{\left[{\left(\frac{1}{\omega_c}\right)}^2+{\left(\frac{d\widehat{A}\left(\omega \right)}{d\omega}\right)}^2\right]}^{\frac{1}{2}} d\omega; \widehat{A}\left(\omega \right)=\frac{A(w)}{A(0)} $$

$$ {\omega}_c\triangleq \min \left\{{\omega}_c^{max},\underset{\omega }{\min}\left\{\left.\omega \right|\widehat{A}(r)<\overline{A},\forall r>\omega \right\}\right\} $$

Where *A*(*ω*) is the Fourier magnitude spectrum of the acceleration signal *a*(*t*), $\widehat{A}\left(\omega \right)$ is the normalized magnitude spectrum.

For SPARC-Acc, $a(t)=\left|\sqrt{a_V^2+{a}_{AP}^2+{a}_{ML}^2}- mean\left({a}_V^2+{a}_{AP}^2+{a}_{ML}^2\right)\right|$ (detrending aims at removing drift from the signal); and similarly, from the gyroscope (SPARC-Gyro), but with no detrending. Direction-wise SPARC measures were also investigated. The same formula were used, but with the values in two axes set to 0; the absolute value was always taken.

### Statistical analysis

Comparisons of subject characteristics were determined using Student t tests for age, height and weight and the Chi square test for categorical variables (i.e., gender). Statistical analyses of the gait measures were adjusted for age, gender, height and weight (using a logistic regression with the logit link function). Comparison between the PD fallers and non-fallers was also adjusted for part III of the UPDRS (logistic regression with the logit link function) [33]. The logistic regression method allows for adjustment for confounding variables while not assuming normality from the data. Fourteen measures (see Table 1) were investigated, therefore, a Bonferroni corrected statistical significance was set at *p* <  0.004, i.e., 0.05/14. In order to compare the degree to which measures differed across groups, effect sizes were used for each measure to quantify the size of the difference between the two groups. For that purpose, Cohen’s *d* was used:

$$ d=\frac{\mu_1-{\mu}_2}{s} $$

Where *d* is the effect size, μ_i_ is the mean for group *i*; *s* is the pooled standard deviation.

$$ s=\sqrt{\frac{\left({n}_1-1\right){s}_1^2+\left({n}_2-1\right){s}_2^2}{n_1+{n}_2-2}} $$

Where *n*_*i*_ and *s*_*i*_ are respectively the mean and standard deviation of group i.

For the comparison of healthy controls and PD, the analysis was repeated with age matched subgroups. The data was normally distributed so *p*-values were calculated using Student t-tests when the two populations had same variance; Welch’s t-tests were used otherwise. Correlations between the values from the PD patients in the OFF condition and the healthy controls were also investigated. The ability of the measures to discriminate PD and control subjects were investigated using Receiver Operating Characteristic (ROC) curves for support vector machine (SVM) analysis performed with 1000 bootstrap replicas. The positive and negative classes were respectively defined as the PD and the controls; the area under the curve (AUC) was used to quantify the discriminative ability of the measures. All the analyses were implemented in MATLAB® (R2015a; MathWorks, Natick, MA).

## Results

Participant characteristics are summarized in Table 2. The control group tended to be older and had a higher percentage of women. Patients with PD were early to mild in the disease course (mean disease duration below 6 years).

**Table 2:** Characteristics of the subjects

	Healthy Controls	Patients with Parkinson’s Disease	*p*-value	Non-fallers	Fallers	*p*-value
# of subjects	39 (19)	101 (24)		64	25	
Age (years)	77.8 ± 3.9 (76.6 ± 1.4)	64.5 ± 9.3 (75.1 ± 2.2)	< 0.001 0.147	63.7 ± 9.8	67.1 ± 9.2	0.14
Gender (% women)	64 (63)	24 (25)	< 0.001 (0.011)	20	0	0.44
Height (cm)	163.1 ± 6.5 (163.0 ± 6.3)	169.4 ± 8.8 (167.1 ± 9.9)	< 0.001 (0.107)	170.7 ± 8.9	168.2 ± 8.8	0.24
Weight (kg)	71.3 ± 13.2 (68.5 ± 12.6)	76.5 ± 11.9 (77.0 ± 10.8)	0.11 (0.030)	75.9 ± 10.5	80.2 ± 14.2	0.12
UPDRS Part III (motor)	0	39.8 ± 12.9 (42.6 ± 11.9)	–	39.8 ± 13.6	41.3 ± 10.6	0.97
Disease duration (years)	0	5.4 ± 3.3 (5.2 ± 2.3)	–	5.1 ± 2.8	6.7 ± 4.4	0.02
Levodopa equivalent dose	0	520.3 ± 314.4 (499.0 ± 300.8)	–	480.0 ± 271.6	582.3 ± 348.0	0.18

Note: Characteristics of the age matched subset of the PD cohort are presented in parenthesis

### Comparison between controls and PD

The SPARC values for accelerometer and gyroscope of the PD patients were generally lower than the controls. Their frequency spectrum compared to controls, once normalized, presents more high amplitude peaks.

**Fig. 1:**
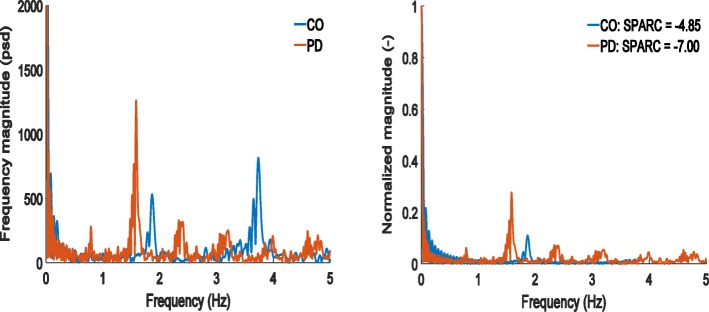
Spectra of a representative PD patient (age: 78 yrs.; disease duration: 7 yrs.; OFF medications) and a healthy control (age: 78). On the left, the un-normalized frequency spectrum is shown. The controls exhibit clear peaks around 1.8 Hz and 3.6 Hz, corresponding respectively to step and stride time. In the PD signal, only a clear peak around 1.5 Hz can be seen. In the normalized frequency spectrum, compared to the control, the spectrum of the PD patient has more peaks with larger amplitude, indicating a less smooth gait

**Fig. 2:**
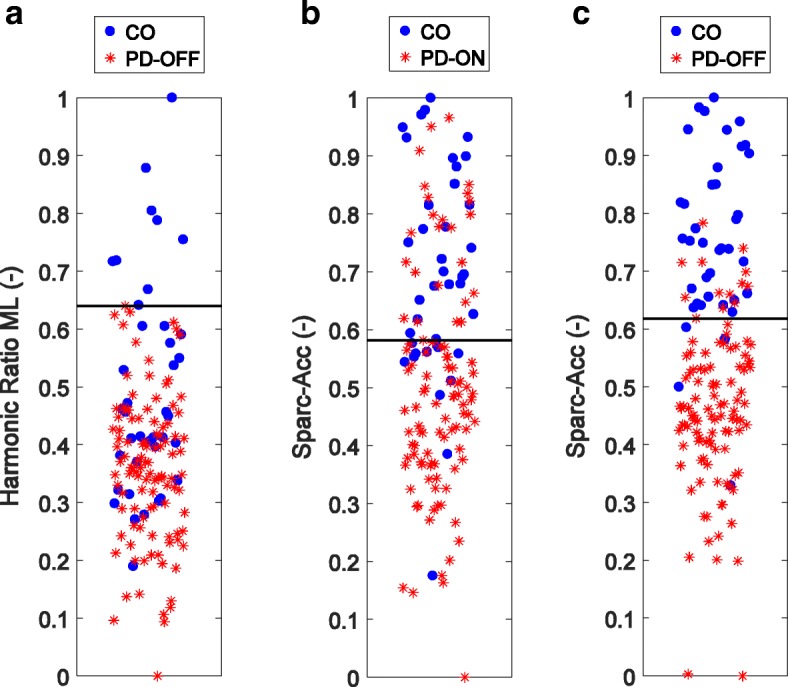
Separation between Controls and PD. **a** Separation from the harmonic ratio in the medio-lateral direction in the OFF state (accuracy: 79%). From SPARC, **b** PD in the ON medication state (accuracy: 74%); **c** PD in the OFF medication state (accuracy: 89%). Gait smoothness in PD decreases sharply in the OFF state. There is a large overlap between the two groups. The horizontal line on each plot is the threshold separating the two groups with the highest accuracy. Note that the horizontal jitter on each plot is meaningless and was added for better visualization

**Fig. 3:**
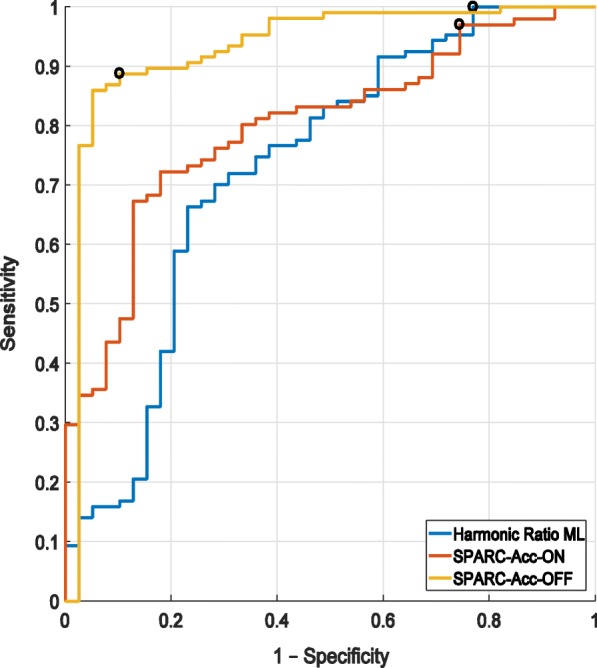
ROC analysis for harmonic ratios ML OFF (blue): AUC = 0.73, SPARC-Acc ON (red): AUC = 0.80 and OFF (yellow): AUC = 0.93. The positive class was defined as the PD. Hence the true positives are controls classified as such while the false positives are healthy controls classified as PD. Classification was done through SVM. Black circles mark optimal thresholds

In the ON medication state, Table 3 shows that SPARC metrics from the accelerometer were significantly lower in PD than in controls (*p* <  0.001). Representative spectra used to compute SPARC are shown in Fig. 1. The harmonic ratio in the medio-lateral direction was also significantly lower in PD than in controls (*p* = 0.002). In the OFF medication state, differences between the PD and the control group increased for most measures (see the effect sizes in Table 3). Importantly, Table 3 and Figs. 2 and 3 show that smoothness quantified by SPARC-Acc has a higher ability for distinguishing controls from PD than harmonic ratios, variability measures or gait velocity; the effect size is largest for the SPARC-Acc measure. Gait speed, harmonic ratios, and SPARC were all significantly differentiate in PD ON versus PD OFF (*p* <  0.005).

**Table 3:** Mean and standard deviation of the gait measures in the two groups

		Healthy Controls (HC)	PD ON	PD OFF	Effect Size & *p*-value		
					HC vs PD ON	HC vs PD OFF	ON vs OFF
SPARC	Acc	-5.17 ± 0.79	−6.11 ± 0.74	−6.74 ± 0.64	**1.25 (< 0.001)**	**2.29 (0.001)**	< 0.001
	V	− 5.08 ± 0.74	− 5.22 ± 0.78	− 5.74 ± 0.88	0.17 (0.107)	**0.78 (0.001)**	< 0.001
	AP	−5.57 ± 0.74	−5.80 ± 0.80	− 6.44 ± 0.77	0.30 (0.190)	**1.14 (0.001)**	< 0.001
	ML	− 5.44 ± 0.32	−5.60 ± 0.48	−5.96 ± 0.50	0.35 (0.388)	1.13 (0.005)	< 0.001
	Gyr	−4.87 ± 0.55	−5.00 ± 0.52	−5.43 ± 0.53	0.25 (0.156)	**1.06 (< 0.001)**	< 0.001
	Yaw	−5.96 ± 0.81	−5.92 ± 0.85	−6.47 ± 0.82	− 0.05 (0.037)	**0.63 (0.002)**	< 0.001
	Roll	− 5.80 ± 0.73	−5.97 ± 0.60	−6.51 ± 0.70	0.26 (0.229)	1.01 (0.007)	< 0.001
	Pitch	−6.28 ± 0.75	−6.55 ± 0.86	− 7.09 ± 0.91	0.32 (0.365)	**0.94 (0.002)**	< 0.001
Harmonic ratios	V	3.10 ± 0.90	2.75 ± 0.82	2.95 ± 0.85	0.42 (0.998)	0.17 (0.092)	< 0.001
	AP	2.08 ± 0.48	2.04 ± 0.74	2.54 ± 0.93	0.06 (0.800)	− 0.55 (0.240)	< 0.001
	ML	0.60 ± 0.11	0.56 ± 0.09	0.52 ± 0.08	**0.33 (0.002)**	**0.89 (< 0.001)**	< 0.001
Mean Gait Speed	–	1.18 ± 0.24	1.16 ± 0.20	1.13 ± 0.19	0.05 (0.028)	0.21 (0.008)	0.117
Stride Time	Mean	1.04 ± 0.06	1.09 ± 0.09	1.09 ± 0.09	− 0.62 (0.048)	− 0.53 (0.365)	0.550
	Variability	3.43 ± 1.17	4.72 ± 3.05	5.84 ± 4.92	− 0.48 (0.044)	− 0.57 (0.055)	0.826

Note: Significant results are displayed in bold

Analysis was also performed without controlling for weight which did not modify the outcomes

As can be seen in Table 4, when the analysis was run on the age matched cohorts, the differences in smoothness, measured through SPARC or harmonic ratios, between groups tended to increase. On the other hand, the differences between the groups in gait speed, mean stride time and stride time variability tended to decrease.

**Table 4:** Mean and standard deviation of the gait measures for the age matched groups

		Healthy Controls (HC)	PD ON	PD OFF	Effect Size & p-value		
					HC vs PD ON	HC vs PD OFF	ON vs OFF
SPARC	Acc	−5.08 ± 0.71	−6.37 ± 0.64	−6.88 ± 0.64	**1.95 (< 0.001)**	**2.69 (0.001)**	0.003
	V	−4.25 ± 0.14	− 4.85 ± 0.61	− 5.77 ± 0.76	**1.36 (< 0.001)**	**2.78 (0.001)**	< 0.001
	AP	−5.42 ± 0.70	−5.83 ± 0.42	− 6.69 ± 0.47	0.73 (0.033)	**2.18 (< 0.001)**	< 0.001
	ML	−5.69 ± 0.56	−5.33 ± 0.43	−5.38 ± 0.49	− 0.73 (0.025)	− 0.61 (0.060)	0.934
	Gyr	− 5.01 ± 0.64	−5.30 ± 0.56	−5.83 ± 0.71	0.49 (0.120)	**1.23 (< 0.001)**	0.009
	Yaw	−5.93 ± 0.82	− 6.14 ± 0.84	− 6.76 ± 0.86	0.26 (0.408)	**1.00 (0.002)**	0.030
	Roll	− 5.91 ± 0.90	− 6.25 ± 0.75	−6.75 ± 0.68	0.41 (0.190)	**1.06 (0.002)**	0.013
	Pitch	−6.30 ± 0.85	−6.77 ± 0.88	−7.61 ± 1.10	0.54 (0.087)	**1.33 (< 0.001)**	0.005
Harmonic ratios	V	2.62 ± 0.65	2.53 ± 0.60	2.99 ± 0.78	0.14 (0.648)	− 0.52 (0.104)	0.010
	AP	1.02 ± 0.46	1.14 ± 0.55	1.98 ± 0.53	− 0.25 (0.424)	**− 0.52 (< 0.001**)	< 0.001
	ML	0.63 ± 0.12	0.57 ± 0.09	0.52 ± 0.06	0.52 (0.100)	**1.18 (0.001)**	0.112
Mean Gait Speed	–	1.20 ± 0.23	1.10 ± 0.20	1.04 ± 0.23	0.48 (0.127)	0.72 (0.026)	0.509
Stride Time	Mean	1.05 ± 0.04	1.12 ± 0.10	1.09 ± 0.12	− 0.87 (0.004)	− 0.43 (0.143)	0.536
	Variability	3.50 ± 1.27	3.67 ± 1.56	5.82 ± 5.05	− 0.12 (0.717)	− 0.066 (0.054)	0.550

Note: Significant results are displayed in bold

### Comparison between PD non-fallers and PD fallers

In the ON state, no significant differences between the fallers and non-fallers could be seen although fairly large effect sizes were observed in the SPARC from the yaw axis (*p* = 0.033, effect size = 0.77) and stride time variability (*p* = 0.010, effect size = − 1.08). In the OFF state, gait performances deteriorated for both the fallers and non-fallers. Significant differences between the two groups could be seen in mean velocity (*p* = 0.001, effect size = 1.18); large effect sizes were observed in SPARC from the vertical axis (*p* = 0.008, effect size = 1.05), yaw (*p* = 0.012, effect size = 0.75) and stride time variability (*p* = 0.006, effect size = − 0.82). ROC analysis was also performed to differentiate the two groups, the best area under the curves was achieved with mean velocity in the off medication case (AUC = 0.80); SPARC from the vertical axis yielded a smaller area (AUC = 0.71).

### Correlation between measures

Correlations between measures are presented in Table 4. SPARC-Acc correlated strongly with the UPDRS motor scores (*r* > 0.60), moderately with the harmonic ratio in the medio-lateral direction, the mean gait speed and the levodopa equivalent dose (0.30 < *r* <  0.50). Correlations between these measures and SPARC-Gyro were usually lower. On the other hand, neither SPARC-Acc nor SPARC-Gyro was significantly related to stride time variability.

## Discussion

To our knowledge, this is the first study to apply the SPARC approach to measure smoothness in gait. Previous work suggested that SPARC was reliable in quantifying smoothness of a reaching task [27]; however, SPARC was not yet applied to quantify smoothness of walking. Our findings support the use of SPARC as a complementary and possibly alternative method to the harmonic ratios commonly used in the literature for quantifying smoothness of gait.

As expected, SPARC was lower in patients with PD than in healthy controls. Similar to previous findings, based on measures from gait variability [13, 34–36] or harmonic ratios [23, 37] the gait of PD subjects was worse than controls. However, SPARC appears to be more accurate in separating the groups. Furthermore, it was previously shown that medication improved patients’ gait [36, 38] and that was also reflected by SPARC which was lower in OFF than ON mediations. Further investigation of medication effects demonstrated that patients in need of larger dose of levodopa tended to have a less smooth gait (*r* = − 0.48), suggesting that these SPARC metrics reflect dopaminergic dysfunction. There was also a strong correlation of SPARC metrics (i.e., accelerometer vector magnitude) with mean gait speed (*r* > 0.5). This makes intuitive sense as an unsmooth gait is associated with and may, perhaps, contribute to slow and unsteady pace. Surprisingly, there was no correlation between SPARC and stride time variability, indicating no linear contribution of smoothness to the rhythm of gait and vice versa. Conversely, SPARC was correlated strongly with the UPDRS motor score (recall Table 5), indicating that smoothness not only is different between PD and controls, but may also provide a general measure of overall motor control ability and its changes across disease severity in PD, at least among ambulatory subjects. In a sense, these findings are parallel to what was shown in patients with stroke: improvements in smoothness in tasks trained during therapy also generalize to movements not explicitly trained as part of the therapy [32].

**Table 5:** Correlations between the gait measures

SPARC Acc	SPARC Acc														
SPARC V	**0.77** (< 0.001)	SPARC V													
SPARC AP	**0.73** (< 0.001)	**0.67** (< 0.001)	SPARC AP												
SPARC ML	**0.70** (< 0.001)	**0.75** (< 0.001)	**0.63** (< 0.001)	SPARC ML											
SPARC Gyr	**0.68** (< 0.001)	0.31 (< 0.001)	0.28 (< 0.001)	0.31 (< 0.001)	SPARC Gyr										
SPARC Yaw	0.54 (< 0.001)	0.38 (< 0.001)	0.28(< 0.001)	0.26 (< 0.001)	0.46 (< 0.001)	SPARC Yaw									
SPARC Roll	**0.64** (< 0.001)	0.49 (< 0.001)	0.28 (< 0.001)	0.42 (< 0.001)	0.58 (< 0.001)	0.32 (< 0.001)	SPARC Roll								
SPARC Pitch	**0.63** (< 0.001)	**0.65** (< 0.001)	0.58 (< 0.001)	0.57 (< 0.001)	0.47 (< 0.001)	0.30 (< 0.001)	0.34 (< 0.001)	SPARC Pitch							
HR V	0.13 (0.033)	0.31 (< 0.001)	0.23 (0.001)	0.25 (0.001)	−0.03 (0.041)	−0.07 (0.216)	0.10 (0.006)	0.21 (0.031)	HR V						
HR AP	− 0.39 (< 0.001)	− 0.20 (0.011)	− 0.24 (0.001)	− 0.25 (< 0.001)	− 0.22 (< 0.001)	−0.06 (0.004)	− 0.22 (< 0.001)	−0.30 (< 0.001)	− 0.02 (0.810)	HR AP					
HR ML	0.36 (< 0.001)	0.32 (< 0.001)	0.34 (< 0.001)	0.20 (0.014)	0.23 (< 0.001)	0.38 (< 0.001)	0.20 (< 0.001)	0.20 (< 0.001)	−0.05 (0.663)	− 0.27 (0.002)	HR ML				
Mean velocity	0.55 (< 0.001)	**0.77** (< 0.001)	0.57 (< 0.001)	0.59 (< 0.001)	0.54 (< 0.001)	**0.66** (< 0.001)	0.57 (< 0.001)	0.46 (< 0.001)	0.32 (< 0.001)	−0.18 (0.032)	0.33 (< 0.001)	Mean velocity			
Stride time Mean	−0.31 (< 0.001)	− 0.45 (< 0.001)	− 0.27 (0.001)	−0.29 (< 0.001)	0.36 (< 0.001)	0.28 (< 0.001)	−0.40 (< 0.001)	−0.23 (0.006)	− 0.09 (0.272)	−0.12 (0.138)	− 0.24 (0.004)	−0.25 (0.002)	Stride time Mean		
Stride time Variability	−0.30 (< 0.001)	− 0.41 (< 0.001)	− 0.26 (0.001)	−0.37 (< 0.001)	−0.29 (< 0.001)	−0.28 (< 0.001)	−0.30 (< 0.001)	−0.20 (0.013)	− 0.22 (0.007)	−0.01 (0.895)	− 0.06 (0.499)	−0.28 (0.001)	0.51 (< 0.001)	Stride time Variability	
UPDRS Motor	**−0.65** (< 0.001)	− 0.35 (< 0.001)	−0.50 (< 0.001)	−0.42 (< 0.001)	−0.41 (< 0.001)	−0.29 (< 0.001)	−0.38 (< 0.001)	−0.42 (< 0.001)	0.02 (0.796)	0.48 (< 0.001)	− 0.29 (< 0.001)	− 0.22 (0.009)	0.10 (0.240)	0.24 (0.004)	UPDRS Motor
LED	−0.48 (< 0.001)	− 0.32 (< 0.001)	− 0.29 (< 0.001)	−0.32 (< 0.001)	− 0.32 (< 0.001)	−0.31 (< 0.001)	−0.34 (< 0.001)	−0.25 (0.002)	− 0.05 (0.531)	0.33 (< 0.001)	− 0.29 (< 0.001)	−0.13 (0.120)	0.18 (0.026)	0.19 (0.023)	0.58 (< 0.001)

Notes: In parenthesis the p-values for the correlations. Absolute correlation |r| > 0.60 displayed in bold

People with PD are more likely to fall than other older adults without PD [16] and are at a greater risk to sustain an injurious fall. PD fallers had significantly decreased smoothness along the yaw direction, in both ON and OFF state, possibly due to bradykinesia and axial rigidity of the trunk [31, 39, 40]. Previous work emphasizes the importance of prospectively assess fall risk [30, 41] and upcoming studies should investigate decreased SPARC as a fall risk marker and further investigate the mechanism underlying the observed changes in this new measure of smoothness.

We demonstrated that gait smoothness could be easily estimated from inertial sensors, which are relatively inexpensive and unobtrusive. Furthermore, data collection is not circumscribed to the laboratory, and further work should explore gait smoothness in real-life settings. For this purpose, SPARC would be particularly suited. It is independent of the duration of the gait bout and, in contrast to stride time variability, does not necessitate identification of fiducial points.

## Conclusions

These initial findings suggest that smoothness of walking is altered in patients with PD. Larger scale and longitudinal studies are needed to further assess the clinical utility of this new approach and to help to study changes in walking in response to specific treatments and interventions.
